# Intranasal Esketamine and Current Suicidal Ideation With Intent in Major Depression Disorder: Beat the Clock, Save a Life, Start a Strategy

**DOI:** 10.3389/fpsyt.2020.00325

**Published:** 2020-04-30

**Authors:** Maurizio Pompili

**Affiliations:** Department of Neuroscience, Mental Health and Sensory Organs, Suicide Prevention Center, Sant’Andrea Hospital, Sapienza University of Rome, Rome, Italy

**Keywords:** suicidal ideation with intent, depression, esketamine, mental pain, hopelessness

## Introduction

While many governments worldwide now consider major depression to be a main cause of disability and provide dedicated programs for treatment improvement, as is done for suicide, the situation seems to be more complicated in terms of assessment and treatment options. Considering depression as a quintessential element of suicide is insufficient due to suicide’s multifactorial nature and clinicians are now requested to provide a critical appraisal of suicide risk, associated or not with major depression (1). Suicide risk does not always receive in-depth analysis when assessing psychiatric patients. Having a diagnosis at hand seems so necessary for paving the way to the next treatment option, suggesting that suicidal or not may ultimately become not so relevant for the therapeutics. On the other hand, being suicidal for some clinicians may be a marker of major depression, and the urgent need to treat such a condition provides the opportunity to explore why suicide risk emerges in a given individual.

The recent innovation in the management of major depression, either resistant to treatment or with suicidal ideation, provides promising opportunities for improving patients’ needs. While antidepressants generally require weeks or even months to work, new options are now available for rapid improvements in depression with suicidal intent (the intent to carry out the suicidal act).

Esketamine is an NMDA receptor antagonist that modulates glutamatergic transmission (2). Recently this drug, available as an intranasal formulation, has been proposed for treatment-resistant depression and for the rapid reduction of symptoms of major depressive disorder in patients with suicidal ideation with intent. According to studies by Daly et al. (3), rapid onset of antidepressant effects was reported after single-dose intranasal esketamine administration to patients with treatment-resistant depression.

Clinicians up to now may have administered antidepressants to suicidal individuals and endorsed such treatment for taking care of individuals in need. From the time of taking antidepressants to the appearance of actual effects, the patients may be experiencing side effects but with no real beneficial effect on the symptoms. Furthermore, the rapid resolution of those components most associated with suicide, such as dysphoria, agitation, irritability, and anxiety, might be totally unaltered by the treatment. Patients may detect when mental pain is overwhelming and report changes in behavior. During the course of a suicidal crisis, the reduction of such suffering is therefore crucial for saving a life.

The new opportunities brought about by recent pharmacological innovations for treating patients with major depressive disorder who have suicidal ideation with intent open up exciting clinical scenarios and a shift in paradigm for the management of such a complex phenomenon. First and foremost, clinicians are now requested to assess suicide risk at all times, a message also delivered by the fifth edition of the *Diagnostic and Statistical Manual of Mental Disorders* (DSM-5) (4), which dedicates a section to suicidal behavior and attention to patient suffering, regardless of the given diagnosis. In my dedicated clinical practice with suicidal individuals, I realized that suicide risk often referred to a specific time frame, which is suggestive of the fact that such risk may be also be reduced if proper intervention is delivered. Of course, in a highly suicidal individual, any intervention may be challenging for saving a life. However, rapid reduction of the pressure resulting from the rumination and inner dialogue which are motivating the wish to die often results in the opportunity to work effectively with these people and change their future.

## Focus on Recent Studies

A small (n = 68) study by Canuso et al. (5) demonstrated a significantly greater improvement in the Montgomery–Åsberg Depression Rating Scale (MADRS) score for the intranasal esketamine group compared to the placebo group at 4 h and ∼24 h, but not at day 25 (patients were treated with either esketamine or placebo in addition to comprehensive standard-of-care treatment). Significantly greater improvement was also observed in the esketamine group on the MADRS “suicidal thoughts” score at 4 h (but not at 24 h or day 25). Patients included in this trial were also assessed with the Suicide Ideation and Behavior Assessment Tool (SIBAT), a newly introduced instrument developed for such a trial. Results from this trial show that overall, in both groups as time passed, the risk of suicide decreased, with most of the subjects progressively being evaluated on the MADRS “suicidal thoughts” item as “enjoying life or take it as it comes” or “weary of life, reporting fleeting suicidal thoughts.” In other words, patients with suicide risk, in most cases, had been moved to a comfort zone with only mild suicidal wishes. These are very promising results for a selected population that, regardless of the type of treatment, received a valuable standard of care for the reduction of suicide risk. Of note is the fact that those receiving placebo plus standard of care also showed a consistent improvement in suicidal ideation during the study period. This was probably due to the fact that patients in the placebo group received the same assistance as patients in the esketamine group. This assistance consisted of medical supervision, assessment of various clinical and physical parameters, and presumably empathic understanding of these patients. Such a trial was also unique as it enrolled patients at imminent risk of suicide, surpassing the exclusion criteria for any other trial except for a few exceptions. It is therefore predictable that the research team and clinicians would have been particularly supportive toward these patients, contributing to the reduction of suicidal ideation by empathic understanding.

Furthermore, two recent large-scale investigations (N=224 and N=227) (6, 7) found that 24 h after the first dose of study medication, esketamine plus comprehensive standard of care (SOC) demonstrated a clinically meaningful and statistically significant reduction of depressive symptoms. Both treatment groups (placebo + SOC and esketamine + SOC) experienced an improvement in the severity of suicidality from baseline to 24 h after the first dose. However, the difference between treatment groups was not statistically significant, possibly due to the interventions towards suicide risk carried out since the beginning of inpatient psychiatric hospitalization. Unlike lithium and clozapine, whose anti-suicidal properties were recognized through observations in clinical practice, esketamine has promised to be a breakthrough since its introduction. Furthermore, lithium, despite considerable evidence, never managed to have official recognition for suicide prevention in mood disorders, whereas clozapine was recognized as being an anti-suicidal treatment for patients suffering from schizophrenia after years of observation and a major dedicated trial (8).

## Paying Attention to the Suicidal Mind

The excitement over a new treatment for depressed patients with suicidal intentions should be re-evaluated after real-world experience. Clinicians should not only assess suicide appropriately but also pay attention to the drama occurring in the patient’s mind by using an empathic approach. Treatment of suicide risk without proper acknowledgment of patient suffering would be counterproductive for many reasons. Suicide ideation or suicide, in general, should not be viewed at the same level as symptoms of depression; instead, it is a complex dimension sharing roots with being depressed. The fact that many depressed patients never think about suicide and have future expectations, even with severe depressive symptoms, should remind clinicians that in those with suicidal ideation, there is an inner private dialogue taking place. Such introspection refers to negative emotions, hopelessness, feelings of despair, or terrible hurt that make life a painful experience. This pain is not generally referred to as being sad or depressed; rather, it emerges from adverse circumstances in one’s life: something missing or gripping the mind that causes both psychic and somatic anguish. When such pain is deemed to be intolerable, suicide is considered as an option in order to escape from such a state. It would, therefore, be reductive to dismiss any suicidal individual without a proper understanding of their suffering.

As a psychiatrist and committed suicidologist, I found a straightforward but extraordinary model useful to understand why an individual wishes to die by suicide. Edwin Shneidman (9) first posited that the individual suicidal experiences unbearable psychological pain (*psychache*) or suffering and that suicide might be, at least in part, an attempt to escape from this suffering. Shneidman (9) considered psychache to be the main ingredient of suicide. According to this model, suicide is an escape from intolerable suffering. Suicide is not a movement toward death but rather an escape from intolerable emotion and unendurable or unacceptable anguish. Experiencing negative emotions, with an internal dialogue making the flow of consciousness painful and leading the individual to the ultimate conclusion, may be related to the fact that if tormented individuals could somehow stop consciousness and still live, they would opt for that solution (10). For Shneidman (11), suicide is the result of an explosive mixture consisting of four basic ingredients: heightened inimicality (acting against the individual’s best interest); exacerbation of perturbation (how disturbed the individual is); increased constriction of intellectual focus; tunneling or narrowing of the mind’s content (dichotomous thinking); and the idea of cessation (the insight that it is possible to stop consciousness and put an end to suffering).

Understanding the suicidal mind requires knowledge of the perturbed state of the individual in crisis, as this provides the motivation for the individual to contemplate suicide. Therefore, asking where the suffering comes from and how it has changed to become more acute is a method of intervention that, although simple and intuitive, is often forgotten by those who are responsible for managing the person in crisis. In the internal debate, essentially involving ambivalence, being able to tune into the suffering of the person makes it possible to stem such ruminations and bring the discussion back to a position of vitality and hope.

Perturbation supplies the motivation for suicide, and lethality is the fatal trigger. Everyone who dies by suicide feels driven to it and feels that suicide is the only option left (10). The concept of “constriction” is defined as tunnel vision or, rather, finding oneself with a reduced number of options to cope with the suffering. Suicidal individuals experience dichotomous thinking, wishing either for some specific (almost magical) total solution to their perturbation or for cessation by suicide. It seems that although there may be effective support from family and friends, the individual is unable to benefit from them.

Proper management of suicide risk should, therefore, consider a shared discussion in which both patient and doctor can explore the origin of suicidal wishes. The vast majority of suicidal patients think about death but also wish to live at the same time. In other words, these individuals want to live as long as there is a reduction in their mental pain. We can achieve the amelioration of such conditions by targeting those symptoms causing more pain and by understanding the patients through active listening, anodyne psychotherapy, and empathic understanding of each patient in their uniqueness as a human being.

## Conclusions

Rapid reduction of suicidal ideation, especially if it is associated with intent, is crucial for saving lives. So far, there have been few pharmacological interventions dedicated to testing drugs relevant to this major clinical challenge. Clinical environments will encounter unprecedented opportunities to assess and manage suicide risk. However, it would be really disappointing if the understanding of why one individual wants to die by suicide is going to be missed. A vast literature is devoted to the subjective experience of patients while they are suicidal, as well as their perception of doctors and nurses during the crises. It would appear that the traditional directive model, with a collection of data, symptoms, etc., is deleterious by creating distance, thus discouraging the crucial alliance between patient and doctor. Patients may perceive such distance as confirmation of being isolated and helpless. Clinicians may also run the risk of a worse manifestation of suicide risk over time if a proper strategy is not adopted. A collaborative approach has been proposed for fostering the alliance between clinician and patient and for in-depth analysis of the components of suicidal wishes (12, 13). Fostering collaboration between patients and clinicians and collecting information through multimodal suicide risk assessment, as well as uncovering suicide risk in the absence of self-disclosure of suicide risk, is, of course, a major goal nowadays (14–16).

There are, of course, many models aimed at describing suicide, as recently reported (17). However, clinicians are encouraged to bridge the gap between theory and practice.

The fact that suicide rates remain mostly unchanged or are on the increase in some parts of the world (18) points to reflections on the paradigms that are more widely embraced in clinical practice. In proposing the phenomenological perspective for understanding suicidal individuals, clinicians can reach the inner and private parts that often allow unlocking of the suicidal mind.

It is worth considering that both ketamine and esketamine may potentially be associated with the risk of addiction. Such possibility should be counterbalanced by the fact that intranasal esketamine is given under strict medical supervision and patients can only receive such a drug in authorized clinics. Besides, it would be misleading to couple the drug discussed in this report with substances of abuse; although of similar origin, the regulatory processes that deliver them to patients are very different. Zhu et al. (19) found that repeated ketamine administration at weekly intervals was found to be safe and effective for maintaining the treatment response as a rapid-acting antidepressant.

There is still also a lack of education and proper information on preventing suicide and how to deal with suicidal individuals. Stigmatization, no doubt, plays a major role in obstructing the adoption of ideas for suicide prevention. The introduction of a new treatment specifically involving the assessment and management of suicide risk is a breakthrough innovation that paves the way a new era of the reduction of depressive symptoms in adults with major depressive disorder who have current suicidal ideation with intent. Clinicians will receive updates and continuous medical education, addressing the difficulties with suicidal individuals. Let us hope that such difficulties become opportunities for unlocking the suicidal mind, getting to know the drama occurring in the minds of these people, and, ultimately, facilitating their wish to live.

## Author Contributions

The author confirms being the sole contributor of this work and has approved it for publication.

## Conflict of Interest

MP took part in advisory boards on esketamine and received consultation fees by Janssen, which are unrelated to this article. In the last 2 years, he has received lectures or advisory board honoraria or engaged in clinical trial activities with Angelini, Lundbeck, Janssen, Otsuka, and Allergan, which are unrelated to this article.
